# Flogging in the British Army

**Published:** 1846-10

**Authors:** 


*Flogging in the British Army.* From Marshall's Military Miscellany.—Our author has fully described the manner in which the various military punishments, including flogging, are carried into effect in our army. As a matter of course, we must refer the curious reader for details to the original. All that we shall do is to pick out a few passages, that may be supposed to have some interest in a medical point of view. When a man is to be flogged, he is, after stripping himself to the waist, tied to a machine termed a triangle, his hands being pulled up to the top of it and there secured; cords are passed round the upper part of the thighs, and the ankles. At other times, the delinquent is lashed to a gun-wheel or a tree. The amount of suffering from the punishment then inflicted may depend a good deal upon the manner in which the prisoner is secured. If the ligatures are too tight, the hands may become quite black and benumbed—the numbness continuing for several days—from the stoppage of the circulation; this arises partly from the man's hanging on, as it were, by the hands. If, again, they be too loose, he is liable to move and wriggle about from side to side, by which means the *cat* falls on unsuitable parts, as the ribs, the neck, or even the face and eyes.

During the last war it was very common to flog upon the breech, and sometimes on the calves of the legs, when the back and breech were unsound from former inflictions. Flogging on the breech is said to be more painful than on the back, probably in consequence of the greater sensibility of the extreme parts of the body.

The first stroke of the cat, says Dr. Marshall, occasions an instantaneous discoloration of the skin from effused blood, the back appearing as if it was thickly sprinkled with strong coffee, even before the second stroke. Sometimes the blood flows copiously by the time the first fifty or one hundred lashes are inflicted; at other times, little or no blood appears when two hundred lashes have been inflicted. During the first one hundred and fifty or two hundred lashes, a man commonly appears to suffer much, considerably more, indeed, than during the subsequent part of a punishment, however large it may be. The effused blood in the skin, or, perhaps, some disorganization of the nerves of sensation, seem to occasion a blunting of its sensibility, and thereby lessen the acuteness of the pain arising from the application of the cat. Left-handed drummers, whose cats are applied to a portion of sound skin, and drummers who have not been sufficiently drilled to flogging, spread the lashes unnecessarily, and excite an unusual degree of pain. Delinquents frequently call out to the drummer to strike higher, then lower, and sometimes alternately.—*Med. Chir. Rev.*

